# Supplementary figures and images for: Identification of hub genes and biological pathways in hepatocellular carcinoma by integrated bioinformatics analysis
Source: PeerJ. 2021 Jan 19;9:e10594. doi: 10.7717/peerj.10594 (PMC7821758; doi:10.7717/peerj.10594)

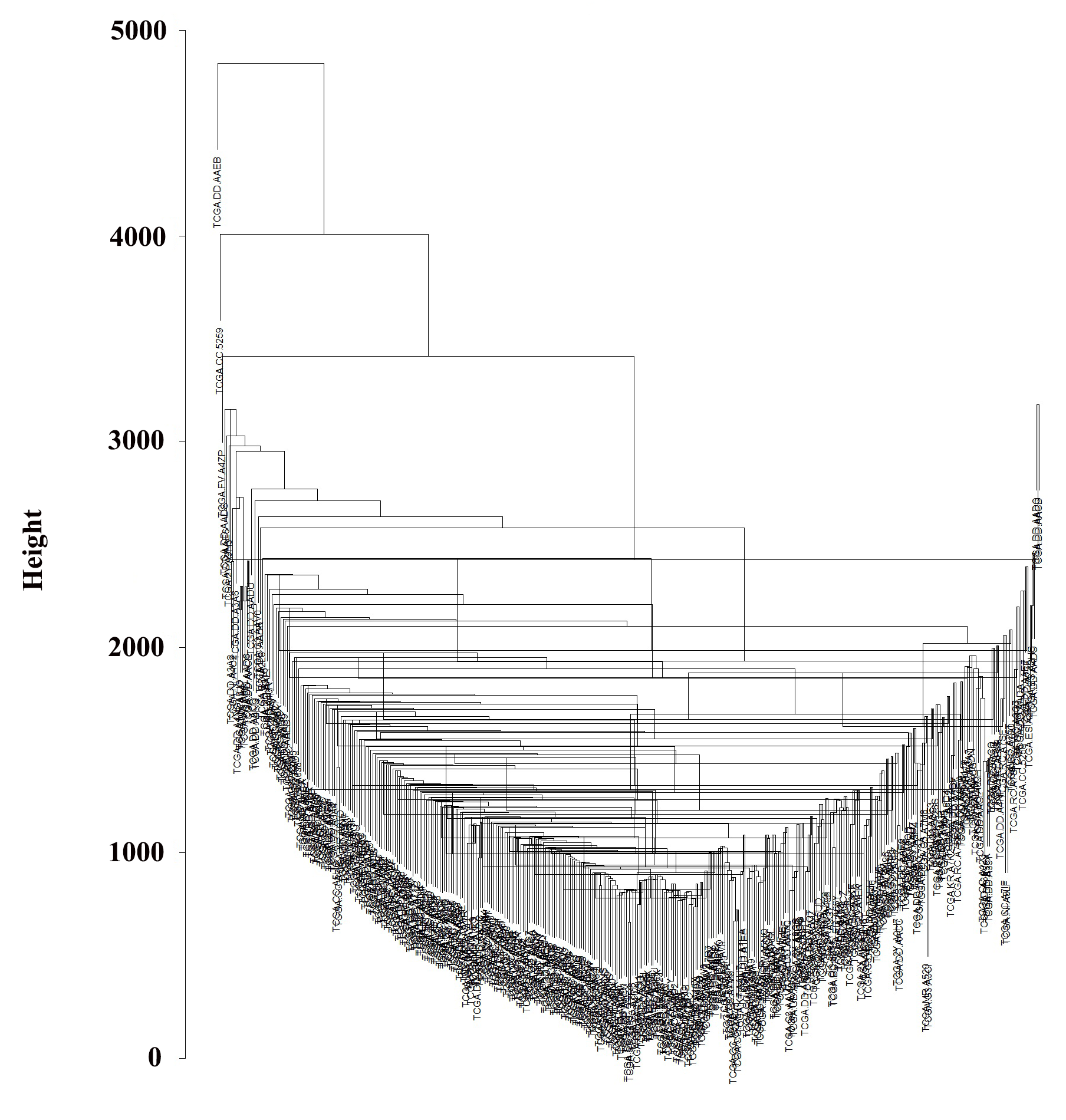

Supplement: Supplemental Information 1 — Sample clustering was conducted to detect outliers, whlie TCGA-DD-AAEB, TCGA-CC-5259 and TCGA-FV-A4ZP were removed. [file peerj-09-10594-s001.png]
